# Influence of antenatal physical exercise on haemodynamics in pregnant women: a flexible randomisation approach

**DOI:** 10.1186/s12884-015-0620-2

**Published:** 2015-08-22

**Authors:** Rhiannon Emma Carpenter, Simon J. Emery, Orhan Uzun, Lindsay A. D’Silva, Michael J. Lewis

**Affiliations:** College of Engineering, Swansea University, Talbot Building, Singleton Park, Swansea, SA2 8PP UK; Department of Gynaecology, Singleton Hospital, Sketty Lane, Sketty, Swansea, SA2 8QA UK; Department of Paediatric Cardiology, University Hospital of Wales, Heath Park, Cardiff, CF14 4XW UK; College of Medicine, Swansea University, Talbot Building, Singleton Park, Swansea, SA2 8PP UK

## Abstract

**Background:**

Normal pregnancy is associated with marked changes in haemodynamic function, however the influence and potential benefits of antenatal physical exercise at different stages of pregnancy and postpartum remain unclear. The aim of this study was therefore to characterise the influence of regular physical exercise on haemodynamic variables at different stages of pregnancy and also in the postpartum period.

**Methods:**

Fifity healthy pregnant women were recruited and randomly assigned (2 × 2 × 2 design) to a land or water-based exercise group or a control group. Exercising groups attended weekly classes from the 20th week of pregnancy onwards. Haemodynamic assessments (heart rate, cardiac output, stroke volume, total peripheral resistance, systolic and diastolic blood pressure and end diastolic index) were performed using the Task Force haemodynamic monitor at 12–16, 26–28, 34–36 and 12 weeks following birth, during a protocol including postural manoeurvres (supine and standing) and light exercise.

**Results:**

In response to an acute bout of exercise in the postpartum period, stroke volume and end diastolic index were greater in the exercise group than the non-exercising control group (*p* = 0.041 and *p* = 0.028 respectively). Total peripheral resistance and diastolic blood pressure were also lower (*p* = 0.015 and *p* = 0.007, respectively) in the exercise group. Diastolic blood pressure was lower in the exercise group during the second trimester (*p* = 0.030).

**Conclusions:**

Antenatal exercise does not appear to substantially alter maternal physiology with advancing gestation, speculating that the already vast changes in maternal physiology mask the influences of antenatal exercise, however it does appear to result in an improvement in a woman’s haemodynamic function (enhanced ventricular ejection performance and reduced blood pressure) following the end of pregnancy.

**Trial registration:**

ClinicalTrials.gov NCT02503995. Registered 20 July 2015.

## Background

Changes in haemodynamic function during ‘normal’ pregnancy have been relatively well characterised although differences in methodologies have led to some inconsistencies between reported findings. Healthy pregnancy is associated with marked changes in haemodynamic function, with increases in cardiac output (CO) of up to 50 % by late pregnancy [1–3]. However, the temporal patterns of change in heart rate (HR) and stroke volume (SV) that lead to this increase in CO are still being debated [4–9]. Systemic vascular resistance and diastolic blood pressure both decrease during pregnancy, reaching a nadir at around 25 weeks gestation [2, 9] and then gradually increasing until term [7–10], whilst systolic blood pressure remains unchanged [2, 8, 11, 12]. What is far less clear is the influence of antenatal physical exercise and an individual’s ‘training status’ on haemodynamic function in pregnancy.

Exercise training in healthy non-pregnant women results in a lower resting HR due to alterations in autonomic control of the heart subsequent to increases in SV and resting CO and a reduction in systolic blood pressure [13]. Changes in haemodynamic response are usually seen in healthy individuals within 3 to 12 weeks of starting an exercise training programme [13, 14] and display a dose–response relationship [15]. The Royal College of Obstetricians and Gynaecologists (RCOG) [16] currently recommend that previously sedentary women should begin with 15 min of continuous exercise three times each week, increasing gradually to 30 min four times each week, and thereafter daily. However, the specific type of exercise required to provoke a sustained change in cardiovascular function during pregnancy has not been determined. It is also highly debatable whether the RCOG guidelines are realistic in terms of likely adherence by pregnant women, and a more pragmatic approach to exercise guidance is needed. Most studies to date have assessed the acute haemodynamic response to a single bout of exercise [17–19] but have not considered the longer-term sustained changes that might result from a training programme and a change in physical fitness. Furthermore, some authors have suggested that the duration of an exercise programme initiated after conception will be too short to result in any significant haemodynamic changes above those already occurring during gestation [20, 21]. Early studies found that resting CO and SV were similar in trained and untrained women during late pregnancy, although resting HR was lower and SV was higher in trained women postpartum [22]. Similar changes were later reported [21], with no significant changes in HR, CO or SV in response to aerobic cycling exercise by late pregnancy, although the pattern of change was altered: peak values for these variables were observed at the end of the second trimester in the non-exercising control group and in the third trimester for the exercise group. These authors speculated that the additional late-pregnancy increase in CO in exercise trained women might be helpful in maintaining venous return and therefore in helping to prevent supine hypotension [21].

The potential benefits of altering haemodynamic function via antenatal exercise training still need to be clarified, but logically an increase in CO and changes in other haemodynamic variables could be advantageous for mother and baby. Further research is now required to more fully assess the haemodynamic changes that occur in response to a programme of antenatal physical exercise. The aim of this study was therefore to characterise the influence of regular physical exercise on haemodynamic variables at different stages of pregnancy and also in the postpartum period.

## Methods

### Participants

Eligible participants were apparently healthy pregnant women aged 18 years or over, with no existing complications of pregnancy at their 12-week dating scan. Participants were recruited (1) through direct contact at the antenatal clinic (during the 12-week dating scan or via telephone), (2) via response to posters placed in the antenatal clinic, local GP surgeries, sports centres and antenatal exercise classes, (3) through advertisements placed on the Health Board website and in local newspapers, and (4) via emails sent to university and hospital staff. Exclusion criteria were: a history of cardiovascular or chronic respiratory problems, sleep apnoea, or central/peripheral nervous system disorder. Individuals who wanted to participate gave their written consent. Participants were informed that they were free to leave the study at any time and this would not affect their standard antenatal care. Ethical approval was obtained from the local (South West Wales) Research Ethics Committee and all procedures were conducted in accordance with the Declaration of Helsinki.

### Study design

**Fig. 1:**
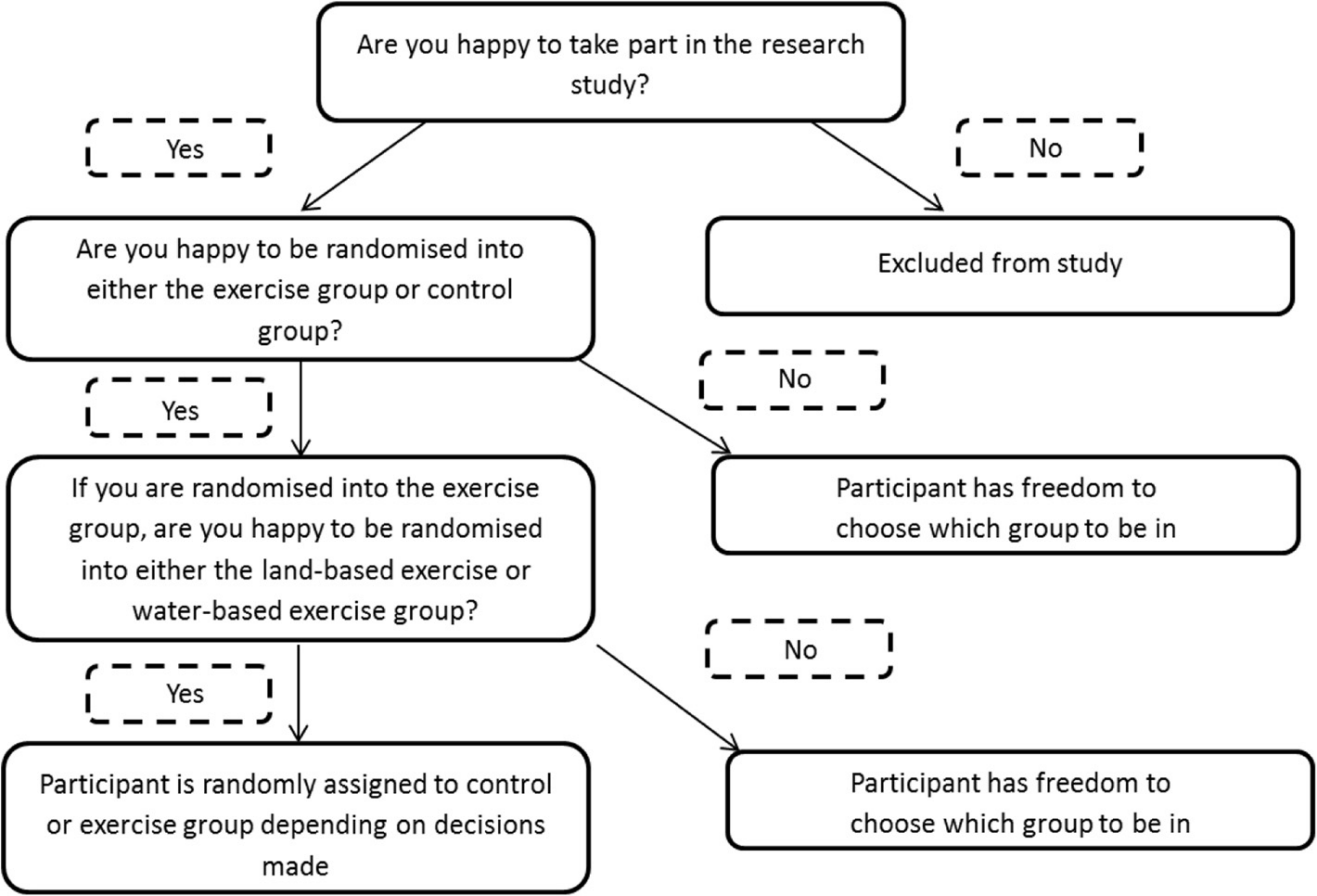
Flow diagram showing the principle of the 2 × 2 × 2 randomised design

Using a 2 × 2 × 2 design [23] participants were randomly assigned to one of three groups: (1) a control group, members of which did not undertake a formal exercise programme, (2) a land-based exercise group, and (3) a water-based exercise group (Fig. 1). Participants were asked a series of questions to determine the group to which they were to be assigned. At each stage they had the option to answer ‘no’ and were free to choose the group to which they preferred to belong.

### Exercise programmes

Participants assigned to the exercise groups started their specific exercise programmes at 20-weeks’ gestation and attended weekly classes until full-term or until they felt they could no longer undertake physical activity. All exercise classes were led or supervised by a qualified midwife. Exercise classes on land and in the water were of similar intensities, assessed via heart rate response. This was continually monitored using heart rate monitors (Polar FT1 Heart Rate Monitor, Polar Electro, Finland; Suunto Memory Belt, Suunto, Finland) and the BORG ‘rating of perceived exertion’ scale [24].

Land exercise classes comprised of 18 min of recumbent cycling, 10 min of stretching and toning exercises and 15 min of pelvic floor exercises. The recumbent cycling exercise (V-Fit BST-RC Recumbent Cycle, Beny Sports Co. UK Ltd., UK) consisted of a 3-min warm-up (with no resistance on the bike) followed by 15-min of continuous cycling. Exercise workload was increased by one ‘level’ on the bike every 2 min, until the participant reached the heart rate target zones for antenatal aerobic exercise suggested by the Royal College of Obstetrics and Gynaecology [16]. Once the target heart rate had been reached, participants were asked to remain exercising at that intensity for 10 min, followed by a cool down period of low resistance cycling to return heart rates to resting values. Water-based exercise classes consisted of a 10-min warm-up followed by 30-min of light-to-moderate intensity ‘aquanatal’ activities such as marching or jogging with various arm actions, weekly throughout pregnancy.

### Physiological measurements

Physiological monitoring was carried out on four occasions: at 12–16, 24–26 and 34–36 weeks gestational age, corresponding to the end of the three trimesters of pregnancy (T1, T2, T3) and also at 12-weeks postpartum (PP). All participants were asked to perform a series of postural manoeuvres and various interventions designed to provoke changes in the cardiovascular and autonomic nervous systems. Participants were asked to refrain from drinking tea, coffee, alcohol or eating a heavy meal within 2 h prior to assessment and to not exercise within 24 h prior to assessment. Anthropometric data for each of the participants was gathered at the start of each measurement session. Weight (Seca digital scales, Seca Ltd., UK) and height (Holtain Stadiometer, Holtain Ltd, UK) were recorded, and used to calculate body mass index (BMI). Two skinfold thickness measurements were taken (Hapenden Skinfold Calipers, British Indicators, West Sussex, UK): one on the bicep and the other on the anterior thigh and two circumference measurements were taken at the wrist and thigh, measured to the nearest 0.1 cm with a flexible tape. These measurements were then used to calculate the change in body fat during pregnancy (Eq. 1) and the body fat mass near term (Eq. 2) [25].

1 $$ \begin{array}{l} Fat\  Change,\  kg\\ {}\begin{array}{l}=0.77\left( weight\  change,\  kg\right)\hfill \\ {} + 0.07\left( change\  in\  thigh\  skinfold\  thickness,\  mm\right) - 6.13\hfill \end{array}\end{array} $$

2 $$ \begin{array}{l} Fat\  mass\  at\  week\ 37,\  kg\\ {}\begin{array}{l}=0.40\left( weight\  at\  week\ 37,\  kg\right)\hfill \\ {} + 0.16\left( biceps\  skinfold\  thickness\  at\  week\ 37,\  mm\right)\hfill \\ {} + 0.15\left( thigh\  skinfold\  thickness\  at\  week\ 37,\  mm\right)\hfill \\ {} - 0.09\left( wrist\  circumference\  at\  week\ 37,\  mm\right)\hfill \\ {} + 0.10\left( prepregnancy\  weight\right) - 6.56\hfill \end{array}\end{array} $$

Participants also completed a Pregnancy Physical Activity Questionnaire (PPAQ) [26] during each of the three antenatal measurement sessions to monitor changes in physical activity as pregnancy progressed. The questionnaire asked the women to record the amount of time they spent completing a number of activities including household chores and care giving (13 activities), work (5 activities), sport and exercise (8 activities), travelling (3 activities) and sedentary activities (3 activities) (Chasan-Taber et al., 2004). The questionnaire took approximately 10 min to complete.

#### Experimental protocol

Participants were first asked to lie in a 45° reclined-supine position for 6 min, after which they were asked to stand for the same duration. Participants then performed a light stepping exercise for 6 min, using the Nintendo Wii games console and ‘balance board’ platform (to provide a visual stimulus for exercise). This was followed by a 6-min seated recovery period. Participants then undertook a 3 min seated cognitive test (to provoke a sympathetic autonomic response), during which they were asked to repeatedly subtract the number 17 from a four digit number (this was performed silently). Participants then breathed synchronously with a metronome for 3 min at a rate of 20 breaths per minute (designed to initiate a parasympathetic response) and then returned to their normal (spontaneous) breathing pattern for 3 min. The total duration of the measurement protocol was 33 min.

#### Physiological variables quantified

Participants underwent continuous Holter ECG monitoring (Pathfinder/Lifecard Digital system; Spacelabs Medical Ltd., UK), providing ECG data with a 1024 Hz sampling frequency. The ECG recordings were assessed for quality by human observation using the Pathfinder system, primarily to verify the absence of excessive noise or artefact. Beat-to-beat cardiac interval (RR) was measured automatically by the Pathfinder system (using a proprietary algorithm) and visually assessed to identify and delete any obvious artefacts (which occurred infrequently, with less than 0.1 % of beats edited in this way). The Task Force Haemodynamic monitor (CNSystems Medizintechnik GMBH, Austria) recorded stroke volume (SV), systolic and diastolic blood pressures (SBP, DBP) on a beat-to-beat basis. The Task Force monitor quantifies SV via transthoracic bioelectrical impedance measurement, in which a small electrical current (<0.4 mA, 40 kHz) is passed into the thorax. This technique has been validated under a variety of conditions against the gold standard (but invasive) thermodilution technique [27] and provides accurate and reliable results. The Task Force monitor also provides continuous non-invasive arterial blood pressure measurement via vascular unloading assessment of the blood pressure in a finger artery. This method provides uninterrupted BP measurement that compares well with intra-arterial BP recordings [28]. The following haemodynamic variables were also quantified from the TFM data: heart rate (HR), cardiac output (CO), total peripheral resistance (TPR), vascular compliance and stiffness, left ventricular ejection time (LVET), end diastolic index (EDI, the end diastolic volume of the left ventricle divided by the body surface area) and cardiac index (CI, the cardiac output divided by the body surface area).

### Statistical analysis

Normality of the data was assessed using the Kolmogorov-Smirnof test. Repeated measures ANOVA with main factors ‘Pregnancy Stage’ (within-group repeated measure) and ‘Exercise Status’ (between-group measure) was used to assess the influence of exercise participation and advancing gestation on the measured physiological variables. Mauchly’s test was consulted to assess the Sphericity of the data; if the assumption of Sphericity was violated then Wilks’ Lambda multivariate tests were used, otherwise Sphericity was assumed. Post-hoc analysis was carried out with Bonferroni correction to identify the locations of significant ‘difference effects’ as appropriate. Independent samples t-tests were also used to assess between group differences at each of the pregnancy stages. Statistical significance was accepted as *p* < 0.05. Effect sizes were quantified as partial eta squared (η^2^). All data are presented as Mean ± SEM (standard error of the mean) and all error bars in the figures represent SEM.

## Results

### Participant characteristics

Fifity women completed all four antenatal assessments, at mean gestational ages of 14.6 ± 1.8, 25.4 ± 1.4 and 34.7 ± 1.6 weeks, and at 13.4 ± 1.8 weeks postpartum. Mean body mass index (BMI) at initial assessment was 24.6 ± 0.7 kg · m^−2^, increasing to 28.2 ± 0.8 kg · m^−2^ by late pregnancy for the control group and 26.4 ± 1.3 kg · m^−2^ increasing to 30.0 ± 1.5 kg · m^−2^ for the exercise group. BMI was not statistically different between groups at either time-point. Fat mass and the change in fat mass between T1 and T3 were not significantly different between the control and exercise groups (*p* = 0.389, *p* = 0.543 rspectively). Participant characteristics and pregnancy outcomes are displayed in Table 1 (a and b). Table 2 shows the activity levels assessed using the Pregnancy Physical Activity Questionnaire (PPAQ) during the first trimester (T1) and third trimester (T3) for the control and exercise groups. Total actvity was not statistically different between the control and exercise groups at either time-point (*p* = 0.070, *p* = 0.089 respectively for T1 and T3). Household activity was significantly higher in the control group during T1 (*p* = 0.004) but not during T3 (*p* = 0.059). Between T1 and T3 there was a significant increase in household activity in the exercise group and a reduction in household activity in the control group (*p* = 0.042). Moderate intensity exercise was significantly higher in the control group during T3 (*p* < 0.0005). Data from the water-based exercise class were excluded from the final statistical analysis owing to recruitment/retention of only a small number of participants in this group (*n* = 4). In the following, ‘Exercise Group’ therefore refers specifically to those participants who took part in the land-based exercise.

**Table 1:** Participant characteristics and pregnancy outcomes

(a)	Control (*n* = 34)		Exercise (*n* = 16)	
	*n*	%	*n*	%
Maternal age at initial measurement (years)				
19–24	1	2.9	3	18.8
25–29	12	35.3	4	25.0
30–34	16	47.1	6	37.5
35–39	5	14.7	2	12.5
40+	0	0	1	6.3
BMI at initial measurement (kg · m^−2^)				
< 18.5	0	0	0	0
18.5–24.9	23	67.6	8	50
25.0–29.9	6	17.6	2	12.5
> 30	5	14.7	6	37.5
BMI at 34 weeks (kg · m^−2^)				
< 18.5	0	0	0	0
18.5–24.9	9	26.5	3	18.8
25.0–29.9	14	41.2	6	37.5
> 30	11	32.4	7	43.8
Planned pregnancy				
Yes	29	85.3	5	31.3
No	3	8.8	9	56.3
Unknown	2	5.9	2	12.5
Parity				
Nulliparous	18	52.9	10	62.5
Primi/Multiparous	16	47.1	6	37.5
Smoking Status				
Previous (prior to pregnancy)	7	20.6	8	50.0
Current	3	8.6	1	6.3
Gestational age at birth (weeks)				
< 34	0	0	0	0
34–36	0	0	0	0
37–40	8	23.5	6	37.5
> 40	26	76.5	10	62.5
Method of delivery				
Vaginal	27	79.4	11	68.8
Caesarean section	7	20.6	5	31.2
Complications				
Breech	0	0	1	0
Prolonged rupture	0	0	1	0
Low platelets	1	0	0	0
(b)	Median	Range	Median	Range
Delivery time (hours:min)^a^				
Total time	4:40	0:27–21:15	4:27	1:05–15:48
1st stage	4:24	0:18–17:09	3:37	0:50–13:20
2nd stage	0:39	0:02–4:02	0:25	0:04–2:47
3rd stage	0:10	0.02–0:25	0:10	0:05–0:22
Birth weight (g)	3490	2620–4820	3470	2780–4340
APGAR score				
1 min	9	4–9	9	4–9
5 min	10	8–10	10	9–10
10 min	10	8–10	10	9–10
Fat change (kg)	1.6	−2.7–11.0	0.35	−3.5–6.4
Fat mass at 35 weeks (kg)	27.1	17.7–40.4	27.8	20.4–43.4

^a^Vaginal delivery group only

*MET* Metabolic Equivalents

**Table 2:** Activity levels (Mean ± SEM) assessed using the Pregnancy Physical Activity Questionnaire (PPAQ) for the control and exercise groups during the first trimester (T1) and third trimester (T3) and the T3 vs. T1 change

Activity	T1			T3			Change (T3-T1)		
	Control	Exercise	p	Control	Exercise	p	Control	Exercise	p
Total activity (MET-h · week^−1^)	354.1 ± 32.9	238.0 ± 34.5	0.070	280.8 ± 37.4	204.7 ± 21.8	0.089	−73.3 ± 54.5	−33.3 ± 17.9	0.371
Sedentary activity (MET-h · week^−1^)	66.6 ± 25.5	88.7 ± 9.5	0.322	57.8 ± 19.4	76.5 ± 8.1	0.300	−8.9 ± 13.3	−12.2 ± 5.0	0.775
Light intensity (MET-h · week^−1^)	124.0 ± 20.2	78.7 ± 13.2	0.086	87.7 ± 18.7	83.7 ± 16.7	0.894	−36.4 ± 28.8	5.0 ± 9.6	0.093
Moderate intensity (MET-h · week^−1^)	156.0 ± 34.7	66.5 ± 26.4	0.080	129.6 ± 20.9	42.7 ± 9.0	<0.0005	−26.5 ± 38.3	−23.8 ± 18.7	0.945
Vigorous intensity (MET-h · week^−1^)	7.5 ± 3.9	4.1 ± 1.5	0.333	5.9 ± 3.4	1.9 ± 1.0	0.311	−1.6 ± 4.4	−2.2 ± 0.9	0.897
Household activity (MET-h · week^−1^)	182.5 ± 43.4	63.0 ± 15.6	0.004	129.8 ± 29.0	67.5 ± 15.5	0.059	−52.8 ± 37.1	4.5 ± 8.4	0.042
Occupational activity (MET-h · week^−1^)	62.3 ± 18.4	93.3 ± 22.5	0.432	44.3 ± 20.2	66.9 ± 8.2	0.288	−18.0 ± 13.8	−26.5 ± 16.9	0.773
Sport/Exercise (MET-h · week^−1^)	30.0 ± 8.4	16.0 ± 6.4	0.244	19.4 ± 8.4	17.1 ± 5.2	0.818	−10.5 ± 8.9	1.1 ± 2.5	0.266

The questionnaire was only completed by a subset of participants (Control, *n* = 5; Exercise, *n* = 14)

*MET* Metabolic Equivalent, *MET*-*h* MET hours, *1 MET* 1 kcal·kg^−1^·h^−1^

### Haemodynamic variables

**Fig. 2:**
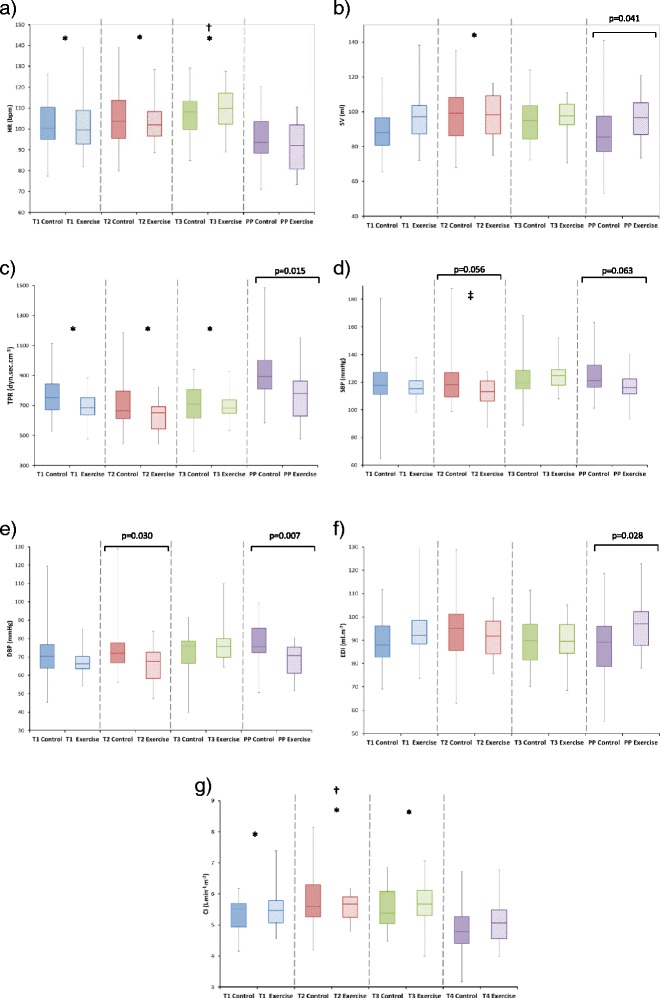
Haemodynamics for control and exercise groups during the ‘exercise’ state for antenatal and postpartum stages (*Statistically different from PP values, † statistically different from T1 values, ‡ statistically different from T3 values; all *p* < 0.05): (**a**) HR, (**b**) SV, (**c**) TPR, (**d**) SBP, (**e**) DBP, (**f**) EDI, (**g**) CI. Pairwise differences identified from post-hoc analysis are also displayed

**Fig. 3:**
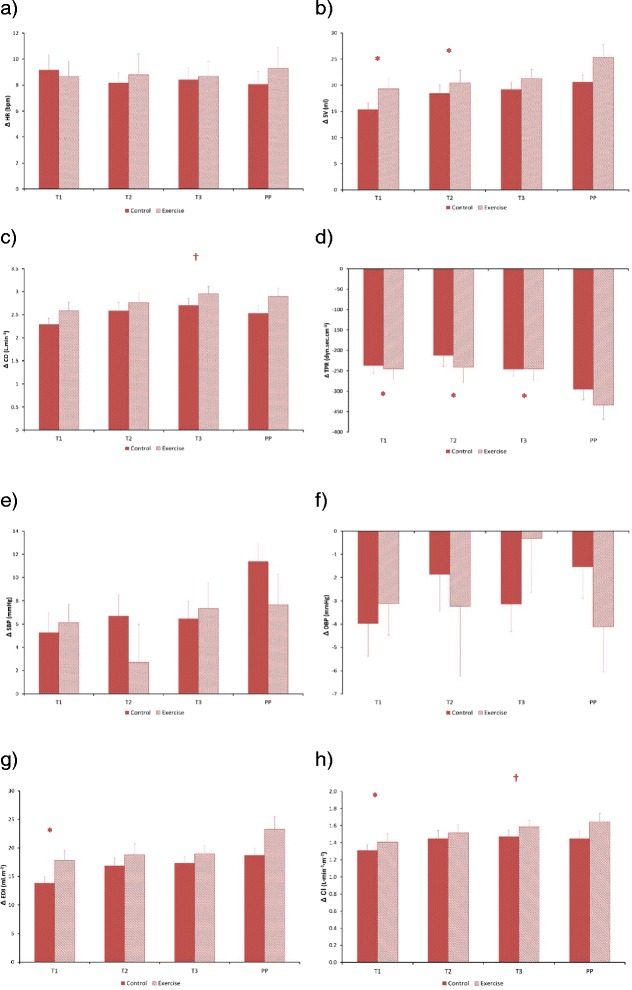
Haemodynamic responses during advancing gestation for control and exercise groups for the standing-to-exercise state change (* Statistically different from PP values, † statistically different from T1 values; all *p* < 0.05): (**a**) ΔHR, (**b**) ΔSV, (**c**) ΔCO, (**d**) ΔTPR, (**e**) ΔSBP, (**f**) ΔDBP, (**g**) ΔEDI, (**h**) ΔCI

**Fig. 4:**
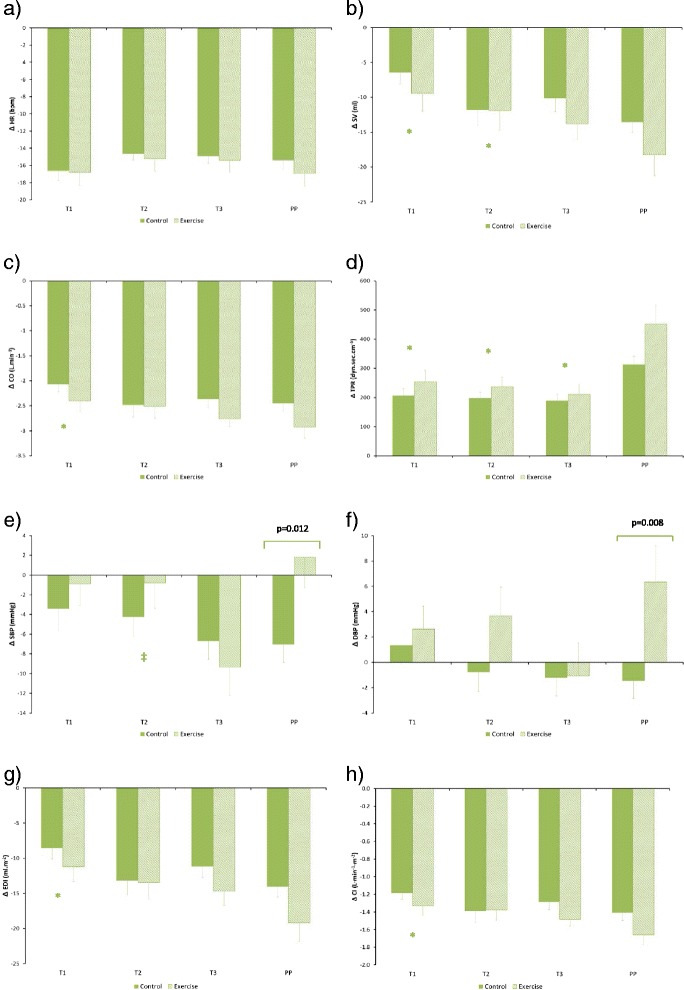
Haemodynamic responses during advancing gestation for control and exercise groups for the exercise-to-recovery change (*Statistically different from PP values, ‡ statistically different from T3 values; all *p* < 0.05): (**a**) ΔHR, (**b**) ΔSV, (**c**) ΔCO, (**d**) ΔTPR, (**e**) ΔSBP, (**f**) ΔDBP, (**g**) ΔEDI, (**h**) ΔCI. Pairwise differences identified from post-hoc analysis are also displayed

As examples of the variation in haemodynamic variables during different physical states, Table 3 shows the values of each of the haemodynamic variables during the supine posture (SUP), standing posture (STA) and supine-to-standing state change (ΔSupSta) for control and exercise groups at each of the pregnancy/postpartum stages. Figures 2, 3 and 4 show a selection of the haemodynamic variables (HR, SV, CO, TPR, SBP, DBP, EDI & CI) as functions of increasing gestation for control and exercise groups during the exercise stage (EXE), during the standing-to-exercise state change (ΔStaExe) and during the exercise-to-recovery state change (ΔExeRec).

**Table 3:** Haemodynamic variables (Mean ± SEM): (a) Supine posture (b) Standing posture and (c) Supine-to-standing between state change for control and exercise groups

Variable	Control				Exercise			
	T1	T2	T3	PP	T1	T2	T3	PP
(a)								
HR (bpm)	80.9 ± 1.5	87.3 ± 1.8	89.1 ± 1.7	74.7 ± 1.7	83.0 ± 3.1	86.6 ± 2.4	94.6 ± 2.5	71.2 ± 2.3
SV (ml)	87.5 ± 2.7	84.1 ± 2.5	76.8 ± 2.1	77.4 ± 2.6	87.3 ± 4.0	83.6 ± 4.1	75.1 ± 3.2	80.7 ± 3.3
CO (L · min^−1^)	7.0 ± 0.2	7.2 ± 0.2	6.8 ± 0.1	5.7 ± 0.2	7.2 ± 0.3	7.1 ± 0.3	7.0 ± 0.3	5.7 ± 0.2
TPR (dyn · sec · cm^−5^)	932.0 ± 32.8	890.9 ± 27.0	985.8 ± 25.9	1118.7 ± 53.0	885.9 ± 42.9	876.1 ± 34.1	956.8 ± 42.7	1122.3 ± 74.5
SBP (mmHg)	109.0 ± 1.7	108.1 ± 1.6	109.6 ± 1.2	108.5 ± 1.6	105.8 ± 1.9	105.1 ± 1.9	111.0 ± 2.4	105.4 ± 2.8
DBP (mmHg)	70.6 ± 1.2	70.1 ± 1.1	72.8 ± 1.2	72.5 ± 1.3	68.0 ± 1.8	67.6 ± 1.6	72.3 ± 2.8	68.6 ± 2.3
EDI (ml · m^−2^)	81.5 ± 2.2	76.7 ± 2.1	70.7 ± 2.0	73.9 ± 1.9	79.7 ± 3.0	73.9 ± 3.1	68.2 ± 2.8	75.7 ± 2.8
CI (L · min^−1^ · m^−2^)	4.1 ± 0.1	4.1 ± 0.1	3.7 ± 0.1	3.3 ± 0.1	4.1 ± 0.2	4.0 ± 0.2	3.8 ± 0.2	3.3 ± 0.2
(b)								
HR (bpm)	92.6 ± 1.7	97.1 ± 2.0	98.4 ± 1.6	87.4 ± 1.8	93.0 ± 3.5	94.8 ± 2.9	100.9 ± 2.6	82.9 ± 2.5
SV (ml)	74.8 ± 1.4	79.5 ± 2.2	75.6 ± 1.9	67.2 ± 1.9	78.3 ± 3.3	78.1 ± 2.3	75.3 ± 2.1	72.5 ± 2.3
CO (L · min^−1^)	6.9 ± 0.1	7.6 ± 0.2	7.4 ± 0.2	5.8 ± 0.2	7.2 ± 0.4	7.3 ± 0.2	7.6 ± 0.3	6.0 ± 0.2
TPR (dyn.sec · cm^−5^)	1002.0 ± 31.5	920.0 ± 43.3	955.5 ± 28.1	1227.9 ± 40.2	930.8 ± 41.4	882.0 ± 32.2	951.3 ± 38.2	1104.8 ± 59.2
SBP (mmHg)	114.1 ± 2.9	114.8 ± 3.0	115.0 ± 1.9	112.9 ± 1.9	111.0 ± 2.3	109.3 ± 2.6	118.3 ± 2.9	109.4 ± 3.0
DBP (mmHg)	76.0 ± 2.2	76.0 ± 2.5	76.0 ± 1.7	78.9 ± 1.4	71.2 ± 1.7	68.9 ± 1.7	76.8 ± 1.9	72.4 ± 2.9
EDI (ml · m^−2^)	75.8 ± 1.4	77.0 ± 1.6	72.5 ± 1.7	69.1 ± 1.6	75.8 ± 3.0	73.2 ± 2.2	70.2 ± 2.3	73.1 ± 2.1
CI (L · min^−1^ · m^−2^)	4.0 ± 0.1	4.3 ± 0.1	4.0 ± 0.1	3.4 ± 0.1	4.1 ± 0.2	4.1 ± 0.1	4.1 ± 0.2	3.4 ± 0.1
(c)								
HR (bpm)	12.1 ± 1.0	9.7 ± 1.1	9.3 ± 1.0	12.7 ± 1.0	10.0 ± 1.3	8.2 ± 1.1	6.3 ± 1.5	11.7 ± 1.0
SV (ml)	−12.7 ± 2.2	−4.6 ± 1.5	−1.2 ± 1.4	−10.2 ± 1.5	−9.1 ± 1.6	−5.4 ± 2.6	0.2 ± 2.3	−8.2 ± 2.5
CO (L · min^−1^)	−0.13 ± 0.17	0.38 ± 0.12	0.61 ± 0.11	0.08 ± 0.09	0.06 ± 0.15	0.20 ± 0.19	0.52 ± 0.16	0.27 ± 0.15
TPR (dyn · sec · cm^−5^)	70.0 ± 22.8	29.1 ± 24.8	−30.3 ± 19.5	39.2 ± 30.9	44.9 ± 22.1	5.9 ± 24.8	−5.6 ± 33.8	−17.5 ± 42.5
SBP (mmHg)	5.1 ± 1.8	6.7 ± 1.9	5.4 ± 1.4	4.4 ± 1.5	5.2 ± 1.2	4.2 ± 2.3	7.3 ± 1.9	4.0 ± 3.1
DBP (mmHg)	5.4 ± 1.4	5.9 ± 1.8	3.3 ± 1.3	6.4 ± 1.5	3.2 ± 1.1	1.3 ± 1.8	4.5 ± 2.1	3.8 ± 2.4
EDI (ml · m^−2^)	−5.7 ± 1.9	0.3 ± 1.2	1.7 ± 1.2	−4.8 ± 1.2	−3.9 ± 1.2	−0.7 ± 2.0	2.1 ± 1.7	−2.6 ± 1.9
CI (L · min^−1^ · m^−2^)	−0.1 ± 0.1	0.2 ± 0.1	0.3 ± 0.1	0.1 ± 0.1	0.1 ± 0.1	0.1 ± 0.1	0.3 ± 0.1	0.1 ± 0.1

On average (across all stages of pregnancy) ANOVA showed that Exercise Status influenced only TPR_EXE_ (*p* = 0.016), DBP_STA_ (*p* = 0.025) and DBP_EXE_ (*p* = 0.028)_._ A significant interaction (Pregnancy Stage x Exercise Status) effect was also observed for SBP_EXE_ (*p* = 0.013), DBP_EXE_ (*p* = 0.005), EDI_STA_ (*p* = 0.010), EDI_EXE_ (*p* = 0.021) and HR_SUP_ (*p* = 0.014)_._ The results of repeated measures ANOVA assessment of the influence of Pregnancy Stage on haemodynamic variables are presented in Table 4.

**Table 4:** Influence of Pregnancy Stage (T1-T3, PP) on haemodynamic variables

Haemodynamic variable	Physical state					
	Supine	Stand	Exercise	ΔSupSta	ΔStaExe	ΔExeRec
HR (bpm)	✓	✓	✓	✓	X	X
	*	*	*	*	*p* = 0.977	*p* = 0.231
SV (ml)	✓	✓		✓	✓	✓
	*	*	*p* = 0.041	*	*p* = 0.001	*p* = 0.001
CO (L.min^−1^)	✓	✓	✓	✓	✓	✓
	*	*	*	*p* = 0.002	*p* = 0.035	*p* = 0.036
TPR (dyn.sec.cm^−5^)	✓	✓	✓	X	✓	✓
	*	*	*	*p* = 0.065	*p* = 0.003	*
SBP (mmHg)	✓	X	✓	X	X	✓
	*p* = 0.032	*p* = 0.117	*p* = 0.028	*p* = 0.749	*p* = 0.122	*p* = 0.026
DBP (mmHg)	✓	X	X	X	X	X
	*p* = 0.037	*p* = 0.216	*p* = 0.075	*p* = 0.857	*p* = 0.788	*p* = 0.156
EDI (ml.m^−2^)	✓	✓	X	✓	✓	✓
	*	*	*p* = 0.300	*	*p* = 0.001	*p* = 0.001
CI (L · min^−1^ · m^−2^)	✓	✓	✓	✓	X	✓
	*	*	*	*p* = 0.004	*p* = 0.058	*p* = 0.049

Separate ANOVA results are shown for each haemodynamic variable during six selected physical states or state-changes. ✓ = Statistical difference between pregnancy stage; X = No statistical difference between pregnancy stage, *p < 0.0005. Pairwise differences identified from post-hoc analysis are discussed in the text

Considering the influence of Pregnancy Stage:

(i) HR_SUP_ increased as pregnancy advanced from T2 onwards (*p* = 0.002, *p* = 0.003 respectively for T2 vs T1 and T3 vs T2). HR_SUP_ also tended to be higher in the exercise group compared to the control group by late pregnancy (*p* = 0.071). HR_STA_ remained unchanged until T2, increasing by late pregnancy (*p* = 0.037). HR_EXE_ was also greater during late pregnancy in comparison to initial measurements (*p* = 0.002). HR_SUP_ (*p* < 0.0005), HR_STA_ (*p* < 0.0005) and HR_EXE_ (*p* < 0.0005) were significantly lower postpartum (PP) than during any of the antenatal measurements. HR_ΔSupSta_ was significantly reduced by late pregnancy (*p* = 0.032), and was reduced at T2 (*p* = 0.003) and T3 (*p* < 0.0005) compared with PP. There was no influence of Pregnancy Stage on HR_StaExe_ or HR_ExeRec._
(ii) SV_SUP_ remained unchanged until T2 and then decreased by late pregnancy (*p* < 0.0005). SV_STA_ was reduced PP in comparison to all antenatal measurements (*p* = 0.001, *p* < 0.0005, *p* = 0.005). There was no influence of gestation on SV_EXE_ but *SV*_*EXE*_*was greater in the exercise group than in the control group PP* (*p* = 0.041). SV_ΔSupSta_ reduced progressively with advancing gestation (*p* = 0.015, *p* = 0.007 for T2 and T3 respectively), and was greater PP in comparison to both T2 (*p* = 0.024) and T3 (*p* < 0.0005). SV_ΔStaExe_ and SV_ΔExeRec_ were also greater PP compared to initial measurements (*p* < 0.0005 and *p* < 0.0005, respectively).
(iii) CO_SUP_ was unchanged with gestation but was higher throughout the antenatal period compared with PP (*p* < 0.0005). CO_STA_ was increased by T2 (*p* = 0.026) and then remained unchanged with advancing gestation. CO_EXE_ was significantly higher by T2 (*p* = 0.011) and T3 (*p* = 0.005) in comparison to initial measurements and all antenatal measurements were greater than PP (*p* < 0.0005). CO_ΔSupSta_ was increased during late pregnancy compared to T1 (*p* = 0.004) and PP measurements (*p* = 0.008). CO_ΔStaExe_ was greater by late pregnancy and CO_ΔExeRec_ was greater PP compared to T1 (*p* = 0.036). *Exercise status did not influence CO in any of the different physical states*.
(iv) TPR_SUP_ remained unchanged until T2 and then increased during late pregnancy (*p* = 0.0002). TPR_SUP_ (*p* < 0.0005, *p* < 0.0005, *p* = 0.001. for T1, T2 and T3 respectively), TPR_STA_ (*p* < 0.0005, all pregnancy stages) and TPR_EXE_ (*p* = 0.002, *p* < 0.0005, *p* < 0.0005) were all lower during pregnancy than PP. *TPR*_*EXE*_*was lower in the exercise group than in the control group PP (p = 0.015).* TPR_ΔStaExe_ (*p* = 0.036, *p* = 0.038, *p* = 0.050, respectively for T1, T2 and T3) and TPR_ΔExeRec_ (*p* < 0.0005, all pregnancy stages) were greater PP than during all antenatal measurements, with a trend towards a greater TPR_ΔExeRec_ response PP in the exercise group (*p* = 0.065).
(v) There was no influence of pregnancy stage on SBP_SUP_, SBP_STA_, SBP_ΔSupSta_ or SBP_ΔStaExe._ Compared with early pregnancy, SBP_EXE_ (*p* = 0.022) and SBP_ΔExeRec_ (*p* = 0.049) were greater from T2 onwards. SBP_EXE_ tended towards a reduction in the exercise group during T2 (*p* = 0.056) and PP (*p* = 0.063). In the exercise group, the SBP_ΔExeRec_ response changed from a reduction to an increase by PP (*p* = 0.012). There was no influence of pregnancy stage on DBP_SUP_, DBP_STA_, DBP_EXE_, DBP_ΔSupSta_, DBP_ΔStaExe_ and DBP_ΔExeRec_. *DBP*_*EXE*_*was significantly lower in the exercise group during T2 (p = 0.030) and PP (p = 0.007)*. As with SBP_ΔExeRec_, the DBP_ΔExeRec_ response changed from a reduction to an increase by PP (*p* = 0.008).
(vi) EDI_SUP_ progressively reduced as pregnancy advanced (*p* = 0.024, *p* = 0.001 respectively for T2 v T1 and T2 vs T3). EDI_STA_ was unchanged until T2, after which it reduced (*p* = 0.006 for T3 vs T1). There was no influence of pregnancy stage on EDI_EXE_, however *EDI*_*EXE*_*was significantly increased PP in the exercise group in comparison to the control group (p = 0.028)*. The EDI_ΔSupSta_ response changed from a reduction to an increase during T2 and T3 when compared to T1 (*p* = 0.027, *p* < 0.0005 respectively), with a similar pattern of change observed when compared to PP (*p* = 0.016, *p* = 0.001). EDI_ΔStaExe_ and EDI_ΔExeRec_ were greater PP than during T1 (*p* = 0.001, *p* < 0.0005 respectively), and both EDI_ΔStaExe_ and EDI_ΔExeRec_ tended to be greater PP in the exercise group (*p* = 0.054 and *p* = 0.073, respectively).
(vii) CI_SUP_ increased after T2 with advancing gestation (*p* = 0.008, *p* = 0.001 respectively for T3 vs T1 and T3 vs T2). CI_STA_ remained unchanged with advancing gestation but was significantly lower PP in comparison to all antenatal measurements (*p* < 0.0005). CI_EXE_ increased until T2 (*p* = 0.041) and then plateaued until the end of pregnancy, and it was lower PP compared to all antenatal measurements (*p* < 0.0005). CI_ΔSupSta_ was increased during late pregnancy (T3) compared to T1 (*p* = 0.001), T2 (*p* = 0.016) and PP (*p* = 0.002). CI_ΔStaExe_ was increased by late pregnancy (*p* = 0.022) and CI_ΔExeRec_ was greater PP compared to T1 (*p* = 0.001). Exercise status did not influence CI in any of the different physical states.

## Discussion

We found that women who had engaged in regular exercise during pregnancy displayed some additional haemodynamic changes compared with non-exercisers: (1) postpartum (PP) values of SV_EXE_ and EDI_EXE_ were greater in the exercise group (*p* = 0.041 and *p* = 0.028, respectively), (2) TPR_EXE_ and DBP_EXE_ were lower in the exercise group postpartum (*p* = 0.015 and *p* = 0.007, respectively) and (3) DBP_EXE_ was also lower in the exercise group during T2 (*p* = 0.030). Thus the main influence of antenatal exercise appears to be an improvement in a woman’s haemodynamic function (enhanced ventricular ejection performance and reduced blood pressure) following the end of pregnancy.

Irrespective of exercise status, pregnant women showed (1) unchanged supine cardiac output (CO) with advancing gestation but higher values during late pregnancy in all other physiological states, (2) increasing heart rate (HR) with advancing gestation (in supine, standing and exercise states) and lower postpartum values, and a lower HR response to standing during late pregnancy, (3) reduced supine stroke volume (SV) and reduced SV response to standing during late pregnancy, (4) increased supine vascular resistance during late pregnancy, and lower vascular resistance (supine, standing and exercise) during pregnancy compared with postpartum values, (5) reduced supine and standing end-diastolic volumes with advancing pregnancy, with an increased end-diastolic response to standing during late pregnancy and increased postpartum responses to exercise and recovery, and (6) increased cardiac index by late pregnancy in the supine posture and in response to standing.

SV behaved as in previous reports, increasing until the start of the second trimester [4, 5, 29] and then either plateauing [8, 30, 31] or declining [3, 7, 9] towards the end of pregnancy. We observed a reduction in SV after T2, although the mechanism behind this change remains unclear. Similarly our observation of HR increasing with pregnancy and peaking in the third trimester are consistent with other studies [2, 7–9, 31, 32]. However, there were some notable differences in the behaviour of CO between our study and previous work. Typically CO has been found to increase during the first trimester [30, 33] and to plateau by the end of the second trimester [7, 30, 33]. An increase in CO of 1 L/min by the 8th week of gestation when compared to pre-conception has been observed [4], with 57 % of the total antenatal increase in CO occurring by 24 weeks’ gestation (typically CO increases by 2.5–3 L.min^−1^ by late pregnancy). Other authors have reported a decline in CO after the 30th week of gestation [8, 9, 32, 34]. In common with us, the majority of these authors used impedance cardiography (ICG) to characterise haemodynamic profiles. It had been claimed that this paradoxical reduction in CO during late pregnancy (physiologically CO would not be expected to decline) reflected the poor technical performance of ICG during this time (anatomical changes in the thorax, due to the enlarging gravid uterus and alterations in maternal body composition were thought to alter the relative electrode configuration and thus directly degrade the ICG signal in pregnant women) [35]. However, as we have demonstrated here and previously [36], when measured in different physiological states (sitting, standing) a physiologically-consistent increase in CO is observed.

During the postpartum period SV_EXE_ and EDI_EXE_ were greater whilst TPR_EXE_ and DBP_EXE_ were reduced in women who had exercised. DBP_EXE_ was also significantly lower in the exercise group during T2. Interestingly however, there were no between-group differences in haemodynamics when measured during the resting state. However, we did not record physical activity levels following pregnancy so we cannot assess whether this might have influenced our postpartum results. In non-pregnant women we would expect a 20-week exercise programme (as performed in our study) to elicit a reduction in resting HR and an increase in both resting SV and CO. However, neither Wolfe et al. (1999) [21] nor Stutzman et al. (2010) [37] found changes in resting HR in response to antenatal exercise (20-week aerobic cycling ergometry exercise programme and a 16-week antenatal walking programme, respectively). There was a suggestion of a continued increase in resting HR into late pregnancy in our exercise group but this was not statistically significant. It therefore appears that maternal HR does not respond to exercise training in the same manner as in non-pregnant women. These previous studies utilised low-to-medium intensity exercise programmes and since changes in physical fitness have a dose–response relationship [15] these may have been insufficient to provoke measurable haemodynamic and heart rate changes [21]. Also it had previously been speculated that an exercise programme beginning after conception would be of too short duration to result in significant haemodynamic change [20], whilst some authors have suggested that the normal physiological changes of pregnancy might be sufficiently dominant to negate the influence of light-to-moderate exercise training on heart rate [21].

Antenatal exercise conditioning has however previously been associated with alterations in the patterns of change in resting HR and SV with advancing gestation, with values peaking in T3 for exercising women and during T2 for controls [21]. Our study did not confirm these findings (we saw similar patterns for both groups) although differences in training protocol could account for this. In Wolfe et al.’s study, participants performed cycle ergometry exercise on 3 days each week at 75 % of age-predicted maximum for 14–25 min, and cardiovascular measurements were recorded at 17, 27 and 37 weeks gestation and postpartum (generally similar to our study).

The present study has extended previous findings to look at the postpartum influence of antenatal exercise on maternal physiology. We observed no difference in resting haemodynamic values during the postpartum period, unlike other authors who reported a reduction in resting HR and an increase in CO in trained compared with untrained women [22]. Wolfe et al. [21] used their postpartum resting measurements only as non-pregnant reference values (for comparison with antenatal measurements) and did not directly compare exercise and control groups post-pregnancy. However, during an acute bout of exercise we observed increases in SV and EDI and reductions in TPR and DBP in the exercise group, suggesting that antenatal exercise improves exercise efficiency during the postpartum period. Interestingly, by late pregnancy the responses to an acute bout of exercise were identical to those of the control group. It would be interesting to investigate if starting an exercise programme at an earlier stage of pregnancy (prior to 12 weeks) might alter the acute response to exercise by mid-pregnancy, and if it might result in more significant haemodynamic changes above those occurring during normal gestation. In the present study, participants did not start exercising until 20 weeks gestation, a time point at which significant haemodynamic adaptation to pregnancy had already occurred. Altering the maternal haemodynamic profile at an earlier stage of pregnancy might be advantageous in reducing the risk of pregnancy-induced diseases such as gestational hypertension and pre-eclampsia.

Similarly it would be of value learn to learn whether exercise still has an advantageous effect postpartum if women stop exercising at the start of the third trimester, or whether exercising during this stage of pregnancy is crucial for post-birth adaptations in maternal fitness. Altering the maternal responses to physical exercise during the postpartum period might be beneficial for mothers in reducing fatigue and improving overall well-being. In particular, from a clinical perspective enhancing the efficiency of exercise during the postpartum period might have a role in protecting women against postpartum cardiomyopathy. Although this remains speculative, such questions could be addressed with larger prospective studies. Future work might also look to examine the influence of continuing exercise during the postpartum period on maternal haemodynamics, and whether it alters the relative rate at which values return to ‘normal’.

We also characterised the between-state changes from standing-to-exercise and exercise-to-recovery. SBP and DBP were significantly altered in the Exercise group when changing from exercise-to-recovery in the postpartum period. Although we cannot comment on the direct significance that this might have on maternal physiology, we speculate that exercise conditioning during pregnancy alters the autonomic nervous system response to these state changes, particularly in terms of blood pressure regulation. However, these findings are based on the overall average for each physiological state (i.e. an average taken over the entire 6 min recording period). We are therefore unable to see the immediate rate of change in blood pressure response post-exercise. In future, it might be useful to look at the beat-to-beat changes in blood pressure to better characterise the dynamic influence that antenatal exercise has on blood pressure control.

## Conclusion

We observed an alteration in cardiovascular response as a result of weekly low-intensity exercise. We are aware that this exercise prescription is below current guidelines for pregnancy, which suggest that previously sedentary women should begin with 15 min of continuous exercise three times a week, increasing gradually to 30 min four times a week and then daily [16]. However, levels of sedentary behaviour are high (particularly in Wales) with 36 % of individuals admitting to performing no weekly exercise [38]. This suggests that a substantial proportion of pregnant women would be unlikely to engage in physical exercise. Women who don’t want to commit to the recommended levels might decide to not exercise at all. If weekly exercise during pregnancy is proven to be sufficient to provide a health benefit then we would argue that more women are likely to engage in this lower level of commitment to antenatal exercise. We speculate that pregnancy might even be used as an opportunity to foster an ethos of exercise amongst previously sedentary women, which might therefore alter behaviours for the rest of their lives.

Our study provides a comprehensive characterisation of haemodynamic responses utilising a randomised control design. The only previous study to implement a controlled experimental design to assess haemodynamic changes during pregnancy in response to antenatal exercise permitted women to choose the group to which they wished to be assigned (exercise or control), thus potentially biasing the outcomes of their study [21]. Our study design enabled a randomised control trial to be performed but still allowed the pregnant women the freedom to choose the intervention group to which they preferred to belong. This flexible randomisation approach therefore encouraged participation amongst women of all physical abilities and minimised the influence of bias on the outcomes of our study.

## Abbreviations

CO: Cardiac output
HR: Heart rate
SV: Stroke volume
RCOG: Royal college of obstetricians and gynaecologists
T1: First trimester
T2: Second trimester
T3: Third trimester
PP: Postpartum
PPAQ: Pregnancy physical activity questionnaire
RR: Cardiac interval
SBP: Systolic blood pressure
DBP: Diastolic blood pressure
TPR: Total peripheral resistance
LVET: Left ventricular ejection time
EDI: End diastolic index
ΔSupSta: Supine-to-standing state changes
ΔStaExe: Standing-to-exercise state changes
ΔExeRec: Exercise-to-recovery state changes
ICG: Impedance cardiography
CI: Cardiac index
